# Curcumin and melphalan cotreatment induces cell cycle arrest and apoptosis in MDA-MB-231 breast cancer cells

**DOI:** 10.1038/s41598-023-40535-5

**Published:** 2023-08-18

**Authors:** Carlos Luan A. Passos, Renata Madureira Polinati, Christian Ferreira, Nathalia Alexia Nascimento dos Santos, Daniel Galinis V. Lima, Jerson Lima da Silva, Eliane Fialho

**Affiliations:** 1 Functional Foods Laboratory, Nutrition Institute, Federal University of Rio de Janeiro, Rio de Janeiro, Brazil; 2 Medical Biochemistry Institute, Federal University of Rio de Janeiro, Rio de Janeiro, Brazil; 3 Departamento de Nutrição Básica e Experimental, Instituto de Nutrição Josué de Castro, Centro de Ciências da Saúde, Universidade Federal do Rio de Janeiro, UFRJ, Cidade Universitária, Ilha do Fundão, Caixa Postal 68041, Rio de Janeiro, CEP 21941-590 Brazil

**Keywords:** Cancer, Breast cancer

## Abstract

Breast cancer is the second most common type of cancer worldwide and the leading cause of cancer death in women. Dietary bioactive compounds may act at different stages of carcinogenesis, including tumor initiation, promotion, and progression. Spices have been used for thousands of years and have many bioactive compounds with chemopreventive and chemotherapeutic properties. Curcumin has a multitude of beneficial biological properties, including anti-inflammatory and anticancer effects. This study investigated the effects of cotreatment with curcumin and the chemotherapeutic drug melphalan in cultured MDA-MB-231 breast cancer cells. When used alone, both curcumin and melphalan had a cytotoxic effect on breast cancer cells. Combined treatment with 11.65 µM of curcumin and 93.95 µM of melphalan (CURC/MEL) reduced cell viability by 28.64% and 72.43% after 24 h and 48 h, respectively. CURC/MEL reduced the number of colony-forming units and increased ROS levels by 1.36-fold. CURC/MEL alter cell cycle progression, induce apoptosis, and upregulate caspases-3, -7, and -9, in MDA-MB-231 cells. Cotreatment with curcumin and melphalan have anti-breast cancer cells effects and represent a promising candidate for clinical testing.

## Introduction

Breast cancer represents a global health challenge. In women, breast cancer is the leading cause of cancer death, and an estimated 2.26 million cases were recorded in 2020^1^. Current treatments include, among others, conventional cytotoxic chemotherapy, but the use of these agents is usually associated with long-term toxicities that may ultimately affect overall survival^2^. The combination of chemotherapeutic agents with chemopreventive phytochemicals has been shown to be a good alternative in the treatment of breast cancer, enhancing the antitumor effect of the conventional chemotherapeutic agents at lower concentrations while attenuating their toxic effects^3,4^. Melphalan is a phenylalanine derivative of nitrogen mustard and an alkylating agent that causing modification and cross linking of DNA, thus inhibiting DNA, RNA and protein synthesis and causing apoptosis in rapidly dividing cells. Its possible hepatotoxic effect is due to the mild and transient elevations in serum aminotransferase levels uncommon with standard doses of melphalan, but occur more commonly with high dose intravenous regimens^5^.

Dietary bioactive compounds have chemopreventive properties as they inhibit proliferation of cancer cells, downregulate the expression of estrogen receptors, and induce cell cycle arrest and apoptosis in tumor cells^6^. McEligot et al. argued that women with a family history of breast or ovarian cancer should be encouraged to adopt a plant-based diet high in fiber, folate, carotenoids, and other anticarcinogenic substances that may prevent these cancers^7^. Moreover, recent research with mammary epithelial cancer cells has demonstrated that their biochemical pathways can be modulated by phytochemicals^8^.

**Figure 1:**
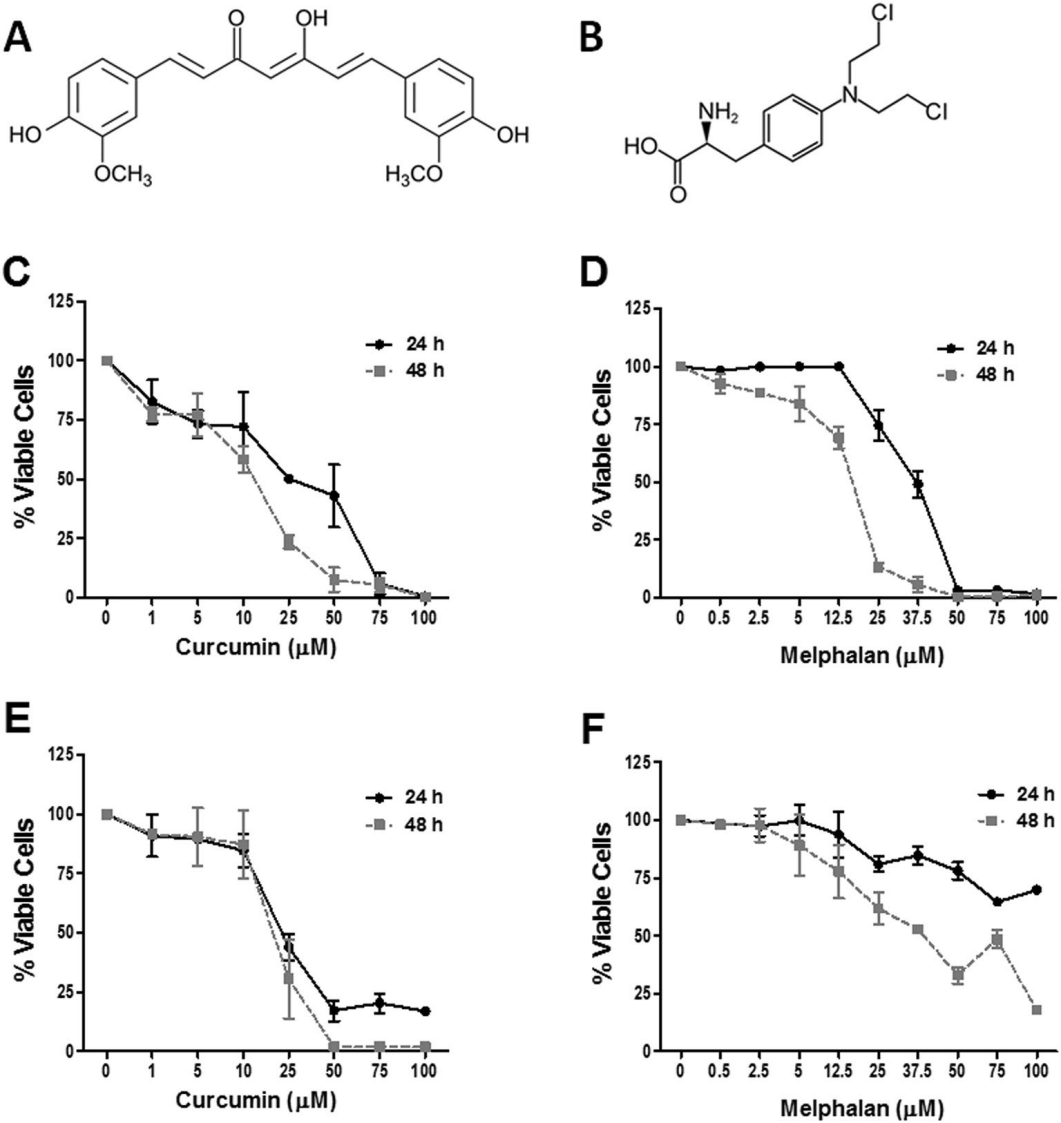
Effect of curcumin and melphalan against breast cancer cell lines MCF-7 and MDA-MB-231. Chemical structure of (**A**) curcumin and (**B**) melphalan. MCF-7 and MDA-MB-231 cells were treated with different concentrations of curcumin (**C**,**E**) and melphalan (**D**,**F**) or control medium for 24 or 48 h and then cell viability was assayed by MTT. Data are expressed as the mean ± SEM of three independent experiments relative to control.

Among functional foods, spices are good candidates to act as preventive agents against non-communicable diseases. Curcumin (Fig. 1A) is the most representative polyphenolic compound extracted from the rhizomes of turmeric (*Curcuma longa* L.), a cultivar member of the Zingiberaceae family widely distributed in tropical and subtropical regions. Turmeric is an economically important genus with diverse uses, such that has long been used for culinary purposes as a spice, food preservative, flavoring agent, and food dye and for various medicinal and cosmetics preparations. Several studies have shown that curcumin affects cellular signaling mechanisms involved in the cell cycle control, apoptosis, proliferation, angiogenesis, metastasis, and inflammation, thus exhibiting therapeutic potential against various cancers^9^. In breast cancer cell lines, curcumin is able to induce apoptosis by transfection of IGFBP-3 resulting in a higher of Bcl-2 family members. Curcumin also induced apoptosis in MCF-7 cells via a p53 dependent pathway^10^. Recently, curcumin has been shown to sensitize carboplatin-resistant triple negative breast cancer cells by inducing increased production of reactive oxygen species (ROS)^11^. However, to date no studies have investigated the cytotoxic effect of curcumin when used in combination with conventional chemotherapy agents such as melphalan (Fig. 1B). Hence, in the present study we firstly analyzed cell viability on human breast epithelial cell line (MCF-10A) and on two breast cancer cells lines (MCF-7 and MDA-MB-231) and assessed the anticancer effects of cotreating MDA-MB-231 breast cancer cells with curcumin and melphalan.

## Results

### Effect of curcumin and melphalan on MCF-7, MDA-MB-231, and MCF-10A cell viability

To evaluate the anticancer potential of curcumin and melphalan, breast cancer cells MCF-7 and MDA-MB-231 and non-tumorigenic human breast cell line MCF-10A were exposed to a range of curcumin or melphalan concentrations for 24 and 48 h and cell viability was assessed by the 3-(4,5-dimethylthiazol-2-yl)-2,5-diphenyltetrazolium bromide (MTT) method. Curcumin (Fig. 1C,E) and melphalan (Fig. 1D,F) had a cytotoxic effect on both breast cancer cell lines in a dose and time-dependent manner. The half maximal inhibitory concentration (IC_50_) values in MCF-7 cells after 24 and 48 h of treatment were 19.85 µM and 11.21 µM for curcumin (Fig. 1C,E) and 33.77 µM and 15.88 µM for melphalan (Fig. 1D,F). In MDA-MB-231 cells, IC_50_ values after 24 and 48 h of treatment were 23.29 µM and 18.62 µM for curcumin and 187.90 µM and 37.78 µM for melphalan, respectively. In addition, IC_50_ values in non-tumor breast cell line MCF-10A after 24 and 48 h of treatment were 187.92 µM and 42.95 µM for curcumin and 195.97 µM and 41.61 µM for melphalan, respectively (Table 1). A selectivity index (SI) was also calculated as the ratio of the IC_50_ from MCF-10A cells and the IC_50_ from MCF-7 or MDA-MB-231 cells. SI values at 24 and 48 h for MCF-7 cells were 9.47 and 3.83 for curcumin and 5.80 and 2.62 for melphalan, respectively. Lastly, SI values for MDA-MB-231 at 24 and 48 h were 8.07 and 2.31 for curcumin and 1.04 and 1.10 for melphalan, respectively.

**Table 1:** Cytotoxicity results of curcumin and melphalan in non-tumorigenic breast cell line MCF-10A and breast cancer cell lines MCF-7 and MDA-MB-231.

Time (h)	Treatment	IC_50_				
		MCF-10A	MCF-7	SI*	MDA-MB-231	SI^#^
24	Curcumin	187.92	19.85	9.47	23.29	8.07
	Melphalan	195.97	33.77	5.80	187.90	1.04
48	Curcumin	42.95	11.21	3.83	18.62	2.31
	Melphalan	41.61	15.88	2.62	37.78	1.10

The selectivity index (SI) was calculated as the IC_50_ ratio of MCF-10A cells to MCF-7 (*) or MDA-MB-231 (^**#**^) cells.

*IC*_*50*_ half maximal inhibitory concentration: the concentration of the tested compound needed to inhibit 50% of the cells.

### Effects of the cotreatment with curcumin + melphalan on MDA-MB-231 cell viability

**Figure 2:**
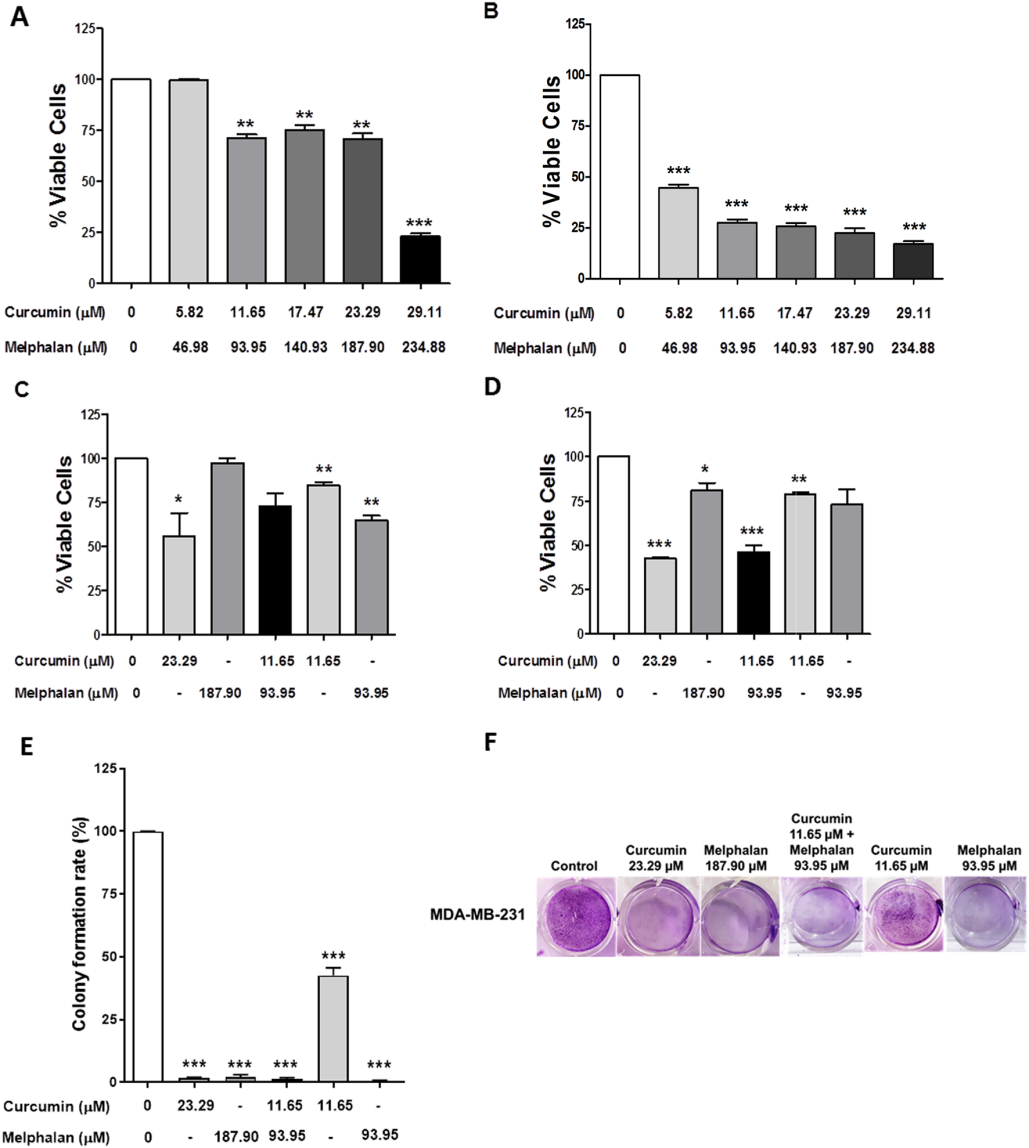
Effect of CURC/MEL cotreatment on MDA-MB-231 cell viability and colony formation. MDA-MB-231 cells were treated with different concentrations of curcumin and melphalan for 24 h (**A**) or 48 h (**B**) and then cell viability was assayed by MTT and Trypan Blue assay for 24 h (**C**) and 48 h (**D**). MDA-MB-231 cells were treated with different concentrations of curcumin, melphalan, CURC/MEL or control medium for 24 h. The number of colonies was determined by crystal violet staining after 18 days of culture (**E**). A representative image is shown (**F**). Data are expressed as the mean ± SEM of three independent experiments. *P < 0.05, **P < 0.001, ***P < 0.0001 relative to control.

Knowing that the use of multiple drugs with different mechanisms or modes of action can direct the effect to a single target or provide a more effective treatment, we tested whether a combined treatment with two compounds could further reduce the viability of breast cancer cells, especially the less sensitive strain MDA-MB-231. Different concentrations of each of the compounds were tested pairwise, taking in consideration their IC_50_ values. For this, concentrations of 0.25, 0.5, 0.75, 1.0, and 1.25-fold of IC_50_ were used. Therefore, MDA-MB-231 cells were treated with 5.82, 11.65, 17.47, 23.29, and 29.11 µM of curcumin in combination with melphalan at concentrations of 46.98, 93.95, 140.93, 187.90, and 234.88 µM, respectively for 24 h (Fig. 2A) and 48 h (Fig. 2B). Cotreatment with 11.65 µM of curcumin and 93.95 µM of melphalan (CURC/MEL) for 24 h and 48 h decreased cell viability by 28.64% and 72.43% (P < 0.05), respectively. Because this combination was the first to show an effect in reducing cell viability after 24 h of treatment, we used the same concentrations in all further experiments with MDA-MB-231 cells. To confirm the cytotoxicity of each compound alone and the combination, MDA-MB-231 cells were treated with each compound at their IC_50_ concentrations (23.29 µM of curcumin or 187.90 µM of melphalan) and at their IC_50_/2 concentrations (11.65 µM of curcumin + 93.95 µM of melphalan), for 24 and 48 h using Trypan blue exclusion assay (Fig. 2C,D). We found that viability of MDA-MB-231 cells treated with 23.29 µM and 11.65 µM of curcumin for 24 h was reduced by 44.15 and 15.5% (P < 0.05 and P < 0.001), respectively. After 48 h, cell viability was reduced by 57.60% (P < 0.0001), 21.34% (P < 0.001), 18.95% (P < 0.05), 26.84%, and 54.0% (P < 0.001) in cells treated with 23.29 µM and 11.65 µM of curcumin, 187.90 µM and 93.95 µM of melphalan, and CURC/MEL, respectively.

### Curcumin, melphalan, and cotreatment with both inhibits clonogenic ability in MDA-MB-231 cells

To further examine the cytotoxic effects of curcumin, melphalan, and CURC/MEL over longer time, clonogenic assays were performed in MDA-MB-231 cells for 18 days. Cells were treated with 23.29 µM and 11.65 µM of curcumin, 187.90 µM and 93.95 µM of melphalan, and also with CURC/MEL for 24 h. Results showed that the clonogenic ability of MDA-MB-231 cells was inhibited by either curcumin or melphalan alone and by CURC/MEL (Fig. 2E,F).

### Effect of curcumin, melphalan, and cotreatment with both on reactive oxygen species (ROS) production

**Figure 3:**
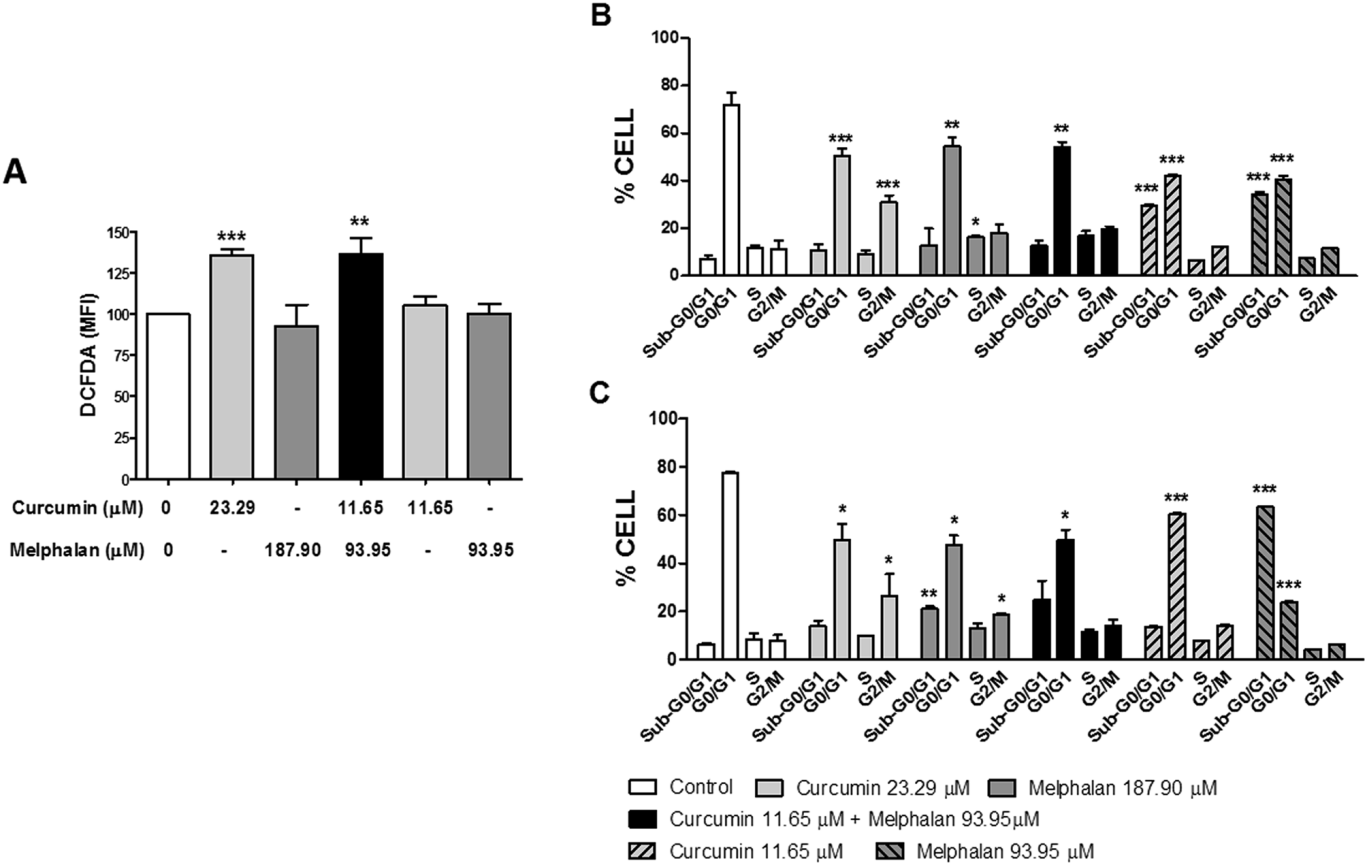
Effect of curcumin, melphalan, and CURC/MEL on reactive oxygen species (ROS) production and cell cycle progression in MDA-MB-231 cells. Detection of ROS (**A**) was performed using MDA-MB-231 cells adhered to 96-well opaque culture plates and treated with curcumin (23.29 µM or 11.65 µM), melphalan (187.90 µM or 93.95 µM), CURC/MEL (11.65 + 93.95 µM) or control medium for 3 h. The cells were then stained with 2′,7′-dichlorofluorescin diacetate and ROS were measured immediately by flow cytometry. MDA-MB-231 cells were treated or not with curcumin (23.29 µM or 11.65 µM), melphalan (187.90 µM or 93.95 µM), CURC/MEL (11.65 + 93.95 µM) or control medium for 24 h (**B**) or 48 h (**C**) and the cell cycle was evaluated after propidium iodide (PI) staining by flow cytometry analysis. The data represent the mean ± SEM of three independent experiments. *P < 0.05, **P < 0.001, ***P < 0.0001 relative to control.

Detection of ROS was performed using MDA-MB-231 cells treated with curcumin, melphalan, and CURC/MEL for 3 h. Treatment with 23.29 µM of curcumin and cotreatment with CURC/MEL increased ROS levels by 1.36-fold in both treatments. However, ROS levels in the cells were not affected by 187.90 µM and 93.95 µM of melphalan and 11.65 µM of curcumin treatment (Fig. 3A).

### Effect of curcumin, melphalan, and cotreatment with both on cell cycle dynamics

In this study, we investigated the effect of curcumin, melphalan, and cotreatment with CURC/MEL for 24 and 48 h on cell cycle progression in MDA-MB-231 cells by PI staining and flow cytometry (Table 2). We found that treatment with 23.29 µM of curcumin for 24 h affected the cell cycle pattern by causing a 1.42-fold reduction in the G0/G1 phase cell population and a 2.8-fold increase in the G2/M phase population. Treatment with 187.90 µM of melphalan for 24 h reduced the G0/G1 phase population by 1.32-fold and increased the S phase population by 1.43-fold. CURC/MEL for 24 h reduced the G0/G1 phase population by 1.33-fold. Treatment with CURC/MEL for 24 h increased in the sub-G0/G1 phase population by 4.19- and 4.81-fold and reduced the G0/G1 phase population by 1.70- and 1.77-fold, respectively (Fig. 3B). Treatment with 23.29 µM of curcumin for 48 h reduced the G0/G1 phase population by 1.55-fold and increased the G2/M phase population by 3.39-fold. In addition, treatment with melphalan for 48 h reduced the G0/G1 phase population by 1.63-fold and increased the sub-G0/G1 and G2/M phase populations by 3.25-fold and 2.38-fold, respectively. Besides, treatment with 11.65 µM of curcumin and 93.95 µM of melphalan for 48 h reduced the G0/G1 phase population by 1.28- and 3.26-fold, respectively, and melphalan was also able to increase in the sub-G0/G1 phase population by 9.76-fold. Lastly, cotreatment with curcumin + melphalan for 48 h reduced the G0/G1 phase population by 1.56-fold (Fig. 3C).

**Table 2:** Percentage of MDA-MB-231 cells in different stages of the cell cycle and type of cell death.

Time (h)	Treatment	% of cells in different e cell cycle phases^#^ (MDA-MB-231)			
		Sub-G0/G1	G0/G1	S	G2/M
24	Control	7.06 ± 0.9	70.50 ± 3.1	11.45 ± 0.8	11.03 ± 2.2
	Curcumin 23.29 µM	10.77 ± 1.5	50.07 ± 1.6***	9.25 ± 1.0	29.87 ± 1.8***
	Melphalan 187.90 µM	12.07 ± 4.1	53.50 ± 2.1**	16.40 ± 0.2*	18.00 ± 2.1
	Curcumin 11.65 µM + Melphalan 93.95 µM	11.86 ± 2.4	53.05 ± 2.2**	16.07 ± 2.1	19.00 ± 1.3
	Curcumin 11.65 µM	29.65 ± 0.5***	42.10 ± 0.4***	6.25 ± 0.1	12.36 ± 0.1
	Melphalan 93.95 µM	34.02 ± 1.1***	40.50 ± 1.7***	7.37 ± 0.2	11.51 ± 0.3
48	Control	6.50 ± 0.4	77.40 ± 0.4	8.30 ± 1.8	7.80 ± 1.8
	Curcumin 23.29 µM	13.85 ± 1.7	49.85 ± 4.5*	9.85 ± 0.1	26.45 ± 6.4*
	Melphalan 187.90 µM	21.15 ± 0.7**	47.50 ± 2.8*	12.80 ± 1.6	18.55 ± 0.4*
	Curcumin 11.65 µM + Melphalan 93.95 µM	24.90 ± 7.9	49.50 ± 4.5*	11.50 ± 0.9	14.05 ± 2.4
	Curcumin 11.65 µM	13.70 ± 0.3	60.25 ± 0.5***	7.94 ± 0.1	14.13 ± 0.3
	Melphalan 93.95 µM	63.46 ± 0.3***	23.74 ± 0.5***	4.34 ± 0.2	6.36 ± 0.2

Time (h)	Treatment	% of cell death^#^ (MDA-MB-231)			
		Necrosis	Apoptosis	Late apoptosis	
24	Control	4.34 ± 0.5	0.79 ± 0.01	1.92 ± 0.3	
	Curcumin 23.29 µM	29.42 ± 5.6***	0.50 ± 0.1*	3.55 ± 0.2*	
	Melphalan 187.90 µM	15.30 ± 2.4**	2.87 ± 0.5**	3.64 ± 0.2**	
	Curcumin 11.65 µM + Melphalan 93.95 µM	17.12 ± 1.0**	2.43 ± 0.1*	3.68 ± 0.04*	
	Curcumin 11.65 µM	5.83 ± 0.4	1.29 ± 0.3	2.39 ± 0.2	
	Melphalan 93.95 µM	7.90 ± 0.2	1.15 ± 0.2	2.66 ± 0.3	
48	Control	5.31 ± 0.1	1.40 ± 0.3	2.44 ± 0.1	
	Curcumin 23.29 µM	18.70 ± 5.3*	0.50 ± 0.3	2.90 ± 0.5	
	Melphalan 187.90 µM	35.40 ± 0.2***	3.05 ± 0.6	5.75 ± 0.9*	
	Curcumin 11.65 µM + Melphalan 93.95 µM	42.40 ± 2.3***	1.60 ± 0.6	5.60 ± 0.4*	
	Curcumin 11.65 µM	3.56 ± 0.9	2.97 ± 0.8	1.43 ± 0.5	
	Melphalan 93.95 µM	7.22 ± 1.8	0.83 ± 0.1	2.70 ± 0.8	

^#^Results are the mean ± SEM of at least three independent experiments.

*P < 0.05, **P < 0.01, and ***P < 0.001 relative to control.

### Effect on cell death of curcumin and melphalan alone, and of curcumin plus melphalan

**Figure 4:**
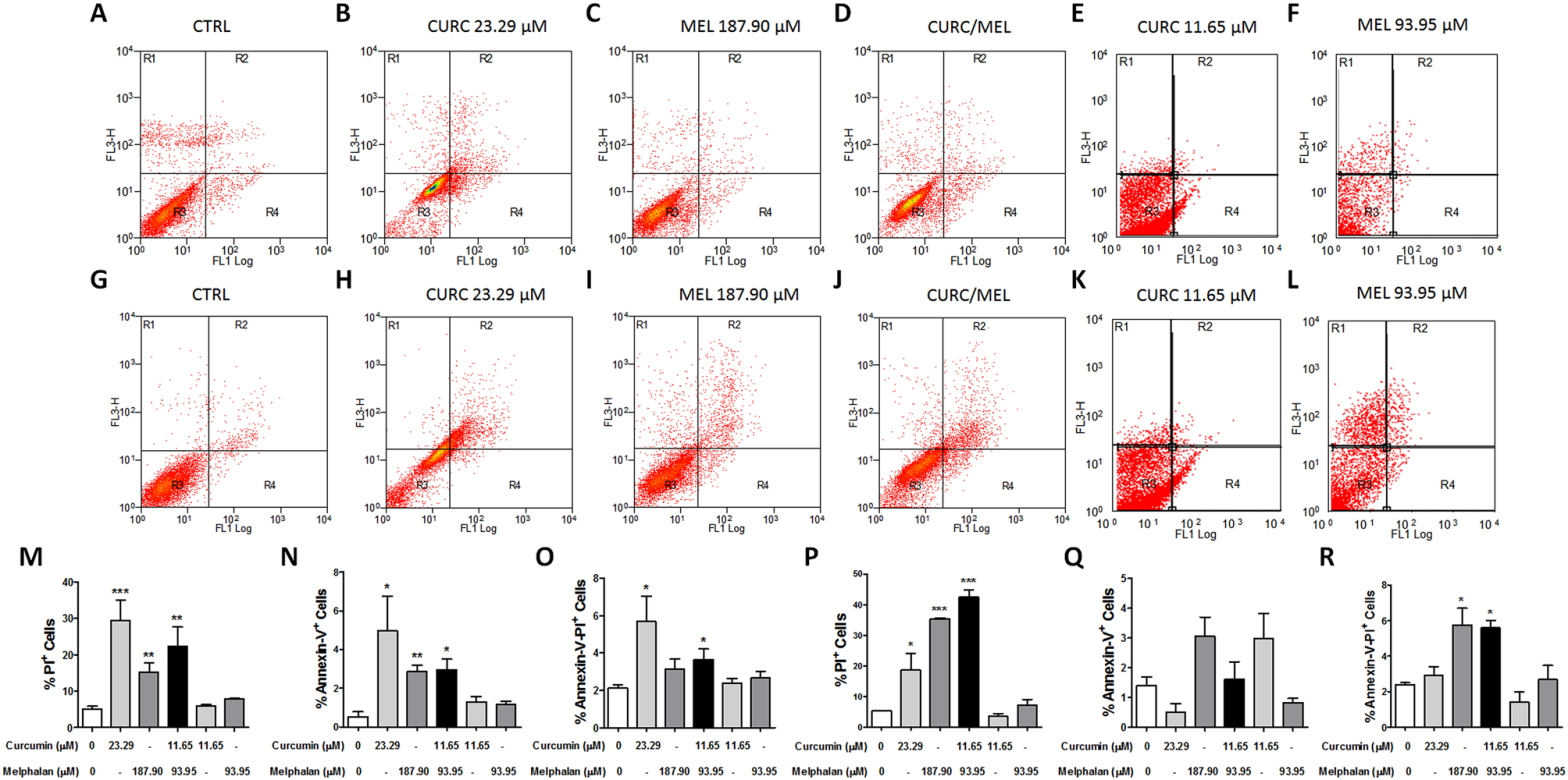
Effect of curcumin, melphalan, and cotreatment with CURC/MEL on type of cell death in MDA-MB-231 cells. The cells were treated with curcumin (23.29 µM or 11.65 µM), melphalan (187.90 µM or 93.95 µM), CURC/MEL (11.65 + 93.95 µM) or control medium for 24 h (**A**–**F**) or 48 h (**G**–**L**). The cells were stained with Annexin V-FITC and propidium iodide (PI), evaluated on a BD FACSCalibur™ flow cytometer (Becton Dickinson) and analyzed using BD CellQuest™ Pro software. PI^+^ cells at 24 h (**M**) and 48 h (**P**), Annexin V^+^ cells at 24 h (**N**) and 48 h (**Q**), and Annexin V/PI^+^ cells at 24 h (**O**) and 48 h (**R**) in MDA-MB-231 cells. The dot plot flow cytometry data represent the mean ± SEM of three independent experiments. *P < 0.05, **P < 0.001, ***P < 0.0001 relative to control.

To discriminate between apoptotic and necrotic cell death, MDA-MB-231 cells were stained with annexin V-FITC and PI and analyzed using flow cytometry (Table 2, Fig. 4). We found that treatment for 24 h with 23.29 µM of curcumin or 187.90 µM of melphalan and combination CURC/MEL increased PI-positive cells ratio, by 5.8, 3.01, and 4.4-fold relative to untreated controls, respectively, which suggests necrotic cell death (Fig. 4A–D,M). In addition, treatment with 23.29 µM of curcumin, 187.90 µM of melphalan, and CURC/MEL for 24 h increased annexin-V-positive cells ratio by 8.5, 5.38, and 5.55-fold, respectively, indicating apoptotic cell death (Fig. 4A–D,N). Also, treatment with 23.29 µM of curcumin and CURC/MEL for 24 h increased annexin-V/PI-positive cells ratio by 2.69- and 1.73-fold, respectively, which indicates late apoptosis (Fig. 4O). Treatment with 23.29 µM of curcumin or 187.90 µM of melphalan and CURC/MEL for 48 h increased PI-positive cells ratio by 3.53-, 6.69-, and 8.01-fold, respectively (Fig. 4G–J,P), but did not affect annexin-V-positive cells ratio (Fig. 4G–J,Q). Lastly, treatment with 187.90 µM of melphalan and cotreatment with CURC/MEL for 48 h increased annexin-V/PI-positive cells ratio by 2.4- and 2.33-fold, respectively (Fig. 4G–J,R). The treatment with 11.65 µM of curcumin or 93.95 µM of melphalan for 24 h and 48 h did not affect PI-positive cells, annexin-V-positive cells and annexin-V/PI-positive cells ratio (Fig. 4E,F,K–L,M–R).

### Effect of curcumin, melphalan, and curcumin plus melphalan cotreatment on p53 and caspases levels

**Figure 5:**
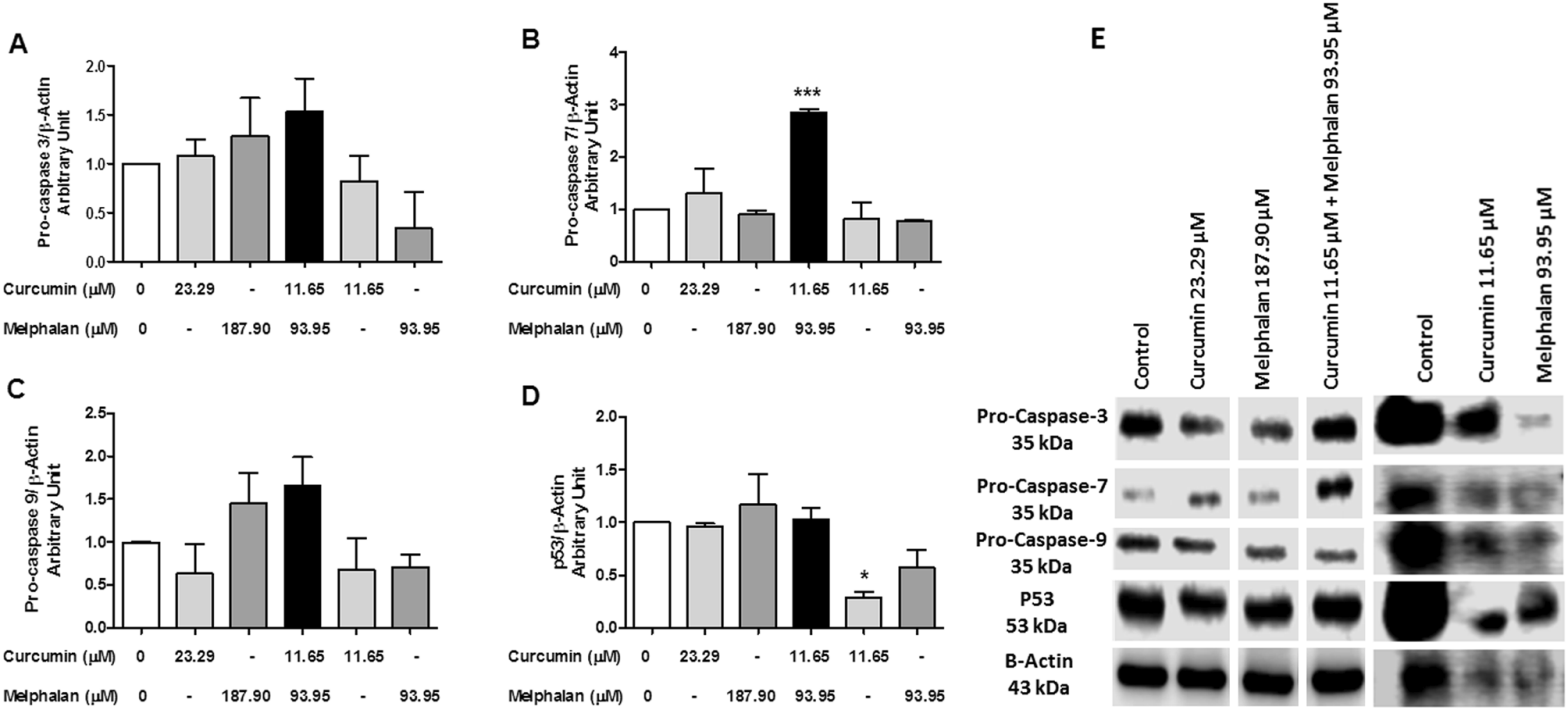
Effect of curcumin, melphalan, and cotreatment with CURC/MEL on p53 levels and activation of caspases in MDA-MB-231 cells. The cells were treated or not with curcumin (23.29 µM or 11.65 µM), melphalan (187.90 µM or 93.95 µM), CURC/MEL (11.65 + 93.95 µM) or control medium for 24 h. Next, equal amounts of total cellular proteins (100 µg) were loaded in each lane for the detection of caspase-3 (**A**), caspase-7 (**B**), caspase-9 (**C**), p53 (**D**), and assayed by Western blotting. Β-actin was used as loading control. The immunoblots (**E**) are representative of three different experiments that gave similar results. White space was used to make explicit for the grouping of blots cropped from different parts of the same gel or from different gels. Densitometric analysis of each lane was calculated using ImageJ software, and the data are expressed as arbitrary units. The data represent the mean ± SEM of three independent experiments. *P < 0.05, ***P < 0.0001 relative to control.

Western blotting analysis determined if curcumin, melphalan, and CURC/MEL affect the levels of p53 and caspases (Fig. 5). Pro-caspase-3 levels increased 1.55-fold in cells treated with CURC/MEL, but this increase was not statistically significant (Fig. 5A). PARP levels were increased by 2.74-, 13.83-, and 2.44-fold after treatment with 23.29 µM of curcumin, 187.90 µM of melphalan, and CURC/MEL for 24 h, respectively (Data not shown). Treatment of MDA-MB-231 cells with CURC/MEL increased the expression of pro-caspase-7 2.88-fold (Fig. 5B). In addition, cotreatment of MDA-MB-231 cells with CURC/MEL increased the expression of pro-caspase-9 1.68-fold (Fig. 5C). However, p53 levels reduced of 3.44-fold after treatment with 11.65 µM of curcumin (Fig. 5D). The combination also increased the expression of p21 1.32-fold in MDA-MB-231 cells (Data not shown). Densitometric analysis of protein bands was conducted to quantify proteins levels (Fig. 5E).

### Effect of curcumin, melphalan, and cotreatment with both on the expression of caspases involved in apoptosis

**Figure 6:**
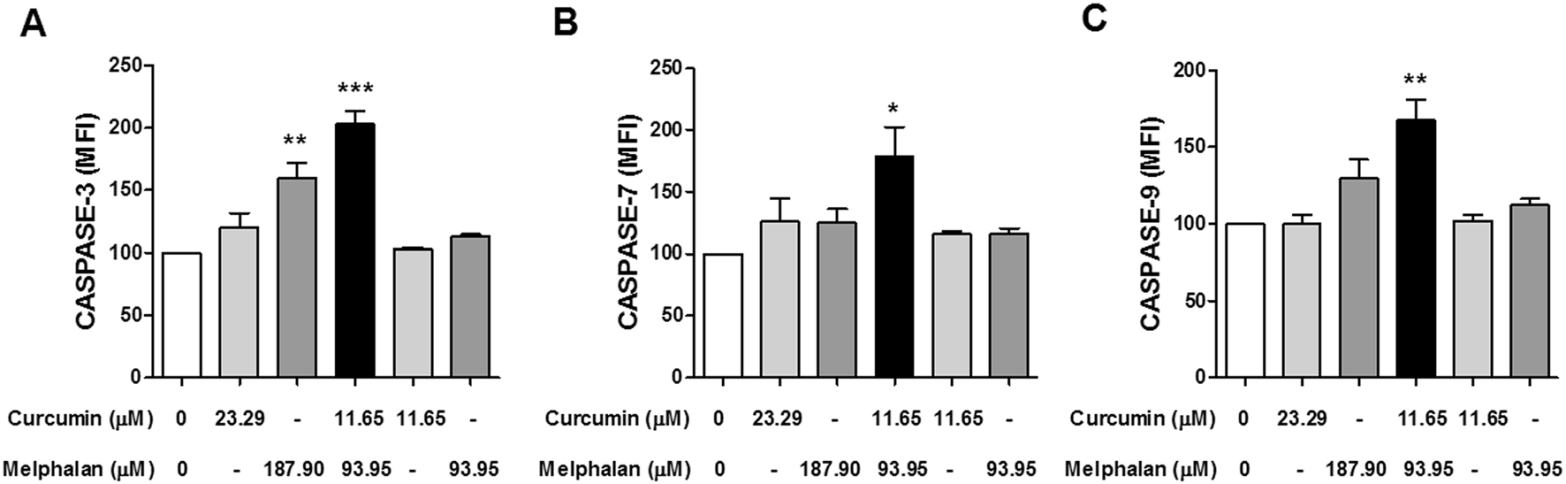
Effect of curcumin, melphalan, and cotreatment with curcumin + melphalan on immunocytochemistry of caspase-3, caspase-7, and caspase-9 in MDA-MB-231 cells. The cells were treated or not with curcumin (23.29 µM or 11.65 µM), melphalan (187.90 µM or 93.95 µM), CURC/MEL (11.65 + 93.95 µM) or control medium for 24 h. The cells were fixed, permeabilized, and stained with primary antibody capase-3 (**A**), caspase-7 (**B**), and caspase-9 (**C**) and then stained with fluorescence secondary antibody. Cell fluorescence was evaluated on a BD FACSCalibur™ flow cytometer (Becton Dickinson) and analyzed using BD CellQuest™ Pro software. The data represent the mean ± SEM of three independent experiments. *P < 0.05, **P < 0.001, ***P < 0.0001 relative to untreated control.

To determine the effect of curcumin, melphalan, and CURC/MEL on MDA-MB-231 cells, levels of caspase-3, caspase-7, and caspase-9 involved in apoptosis were analyzed by immunocytochemistry. We found that CURC/MEL for 24 h increased the levels of caspase-3 (Fig. 6A), caspase-7 (Fig. 6B), and caspase-9 (Fig. 6C) by 2.03-, 1.78-, and 1.67-fold, respectively, compared with untreated controls.

## Discussion

Curcumin is a yellow polyphenol pigment derived from the rhizomes of *C. longa* (turmeric) that has several biological activities^12^. Curcumin has been shown to affect different molecular targets and signaling pathways involved in the development of several cancers^13,14^. Thus, curcumin is a promising candidate as an anticancer drug to be used alone or in combination with conventional cytotoxic drugs^15^. However, to date no study had investigated the effect of curcumin in combination with the cytotoxic agent melphalan. This study is the first to investigate the effects of cotreatment with CURC/MEL on breast cancer cells. We initially tested the cytotoxicity of curcumin and melphalan in MCF-7 and MDA-MB-231 breast cancer cell lines and MCF-10A, a non-tumorigenic breast epithelial cell line. Curcumin and melphalan were shown to have the potential to reduce the viability of both tumor cell lines, but the MCF-7 cell line was the most responsive to the treatments. Hu et al.^16^ demonstrated that curcumin inhibited the proliferation of a variety of breast cancer cell lines after 72 h of treatment. The authors reported that curcumin had greater cytotoxic activity in estrogen receptor-positive cell lines such as MCF-7, T47D, and MDA-MB-415, with IC_50_ values of 2.07 µM, 1.32 µM, and 4.69 µM, respectively. However, curcumin was less active in estrogen, progesterone, and HER2 receptor-negative cell lines such as MDA-MB-231, MDA-MD-468, and BT-20 with IC_50_ values of 11.32 µM, 18.61 µM, and 16.23 µM, respectively. These results are consistent with other studies showing that curcumin inhibits breast cancer cell proliferation but does not affect normal breast epithelial cells^17,18^.

Curcumin was shown to have the potential to reduce the viability of tumor cells while exerting a reduced cytotoxic effect on non-tumorigenic MCF-10A cells. Moreover, the selectivity index (SI) of curcumin to tumor cells showed little potential for adverse effects unlike melphalan, which exhibited lower SI values compared to curcumin, indicating greater toxicity of the conventional chemotherapeutic agent contributing to its adverse effects.

The MDA-MB-231 cell is a triple-negative breast cancer cell line that does not express estrogen receptor, progesterone receptor, or HER2 protein, making therapies for this type of cancer difficult^19^. Because treatment of triple-negative breast cancer cells is challenging, we focused on analyzing the anticancer effects of curcumin in combination with melphalan on these cells. Melphalan is a phenylalanine derivative of nitrogen mustard that exerts its biological activity by inducing interstrand cross-links in DNA^20^. We have previously reported that resveratrol, in association with melphalan, has cytotoxic effects in MCF-7 and MDA-MB-231 cells in vitro^21^. In the current study, we demonstrated that CURC/MEL does not significantly affect cell viability, although levels of PARP, caspase-3, caspase-7, and caspase-9 were enhanced in MDA-MB-231 cells. Zou et al.^22^ showed that curcumin increased the sensitivity of MCF-7 and MDA-MB-231 cells to conventional chemotherapeutic drugs paclitaxel, cisplatin, and doxorubicin. Here, cell membrane integrity was evaluated by the Trypan blue exclusion assay after treatment with curcumin, melphalan, and CURC/MEL. Treatment with curcumin inhibited the growth of 58% of MDA-MB-231 cells after 48 h, and the combination with melphalan inhibited 54% of cells. Similarly, Nyankson et al.^23^ reported that curcumin was able to inhibit 49% of MCF-7 cells using the same assay. Cell membrane disruption was confirmed in the PI and annexin-V assay: cells treated with curcumin or melphalan and CURC/MEL showed a high percentage of cells labeled for PI, which only penetrates the cells when the membrane loses its integrity. Coker-Gurkan et al.^24^ reported that treatment of MDA-MB-231 and MDA-MB-453 cells with curcumin increased the percentage of PI-labeled cells by 5- and 16-fold, respectively, whereas a similar effect was not observed in MCF-7 cells. Moreover, cells treated with curcumin, melphalan, and CURC/MEL showed a high percentage of cells labeled for annexin-V after 24 h of treatment, indicating apoptotic cell death. Chen et al.^25^ showed that curcumin enhanced the antiproliferative effects of doxorubicin in BT-20 triple negative breast cancer cells. Additionally, these authors observed that the combination of curcumin and doxorubicin increased the cleaved forms of PARP and caspase-3, both apoptosis markers. Calaf et al.^26^ found that curcumin and paclitaxel alone and in combination induced cell death by apoptosis and necrosis in MCF-7 and MDA-MB-231 breast cancer cells. Here, CURC/MEL induced apoptosis in the MDA-MB-231 cell line. Importantly, the induction of apoptosis was studied through activation and expression of proteins. The results showed that curcumin, melphalan, and CURC/MEL increased PARP levels and that cotreatment with CURC/MEL increased the levels of caspase-7 and p21. He et al.^27^ reported that curcumin induced apoptosis and cell cycle arrest in MCF-7 breast cancer cells through activation of p21 and cleaved PARP. In the current study, CURC/MEL induced the expression of proteins caspase-3, -7, and -9 involved in apoptosis in MDA-MB-231 cells. These findings suggest that CURC/MEL induces apoptosis in MDA-MB-231 cells through a caspase-3-, caspase-7-, caspase-9-, PARP-, and p21-mediated pathway. Interestingly, caspase-3 is the main effector caspase catalyzing the cleavage of many key cellular proteins within the apoptotic machinery^28^.

## Conclusion

This study investigated the effects of cotreatment with curcumin and melphalan on MDA-MB-231 breast cancer cells. The findings of this experimental study on the combination of phenolic compounds such as curcumin and conventional chemotherapeutic agents such as melphalan have important implications for cancer chemotherapy and their effects should be tested in human clinical trials.

## Methods

### Chemicals

Curcumin (1,7-bis(4-hydroxy-3-methoxyphenyl)-1,6-heptadiene-3,5-dione), melphalan ((2S)-2-amino-3-{4-[bis(2-chloroethyl)amino]phenyl}propanoic acid), and DMSO (dimethyl sulfoxide) were purchased from Sigma-Aldrich (St. Louis, MO, USA). All chemicals used in this study were of analytical grade.

### Cell lines and cultures

The human breast epithelial cell lines MDA-MB-231, an estrogen receptor-negative cell line derived from a metastatic carcinoma; MCF-7, an estrogen receptor-positive cell line derived from an in situ carcinoma; and MCF-10A, a non-tumorigenic breast cell line, all obtained from American Type Culture Collection (ATCC; Manassas, VA, USA), were used in this study. MDA-MB-231 and MCF-7 cells were maintained in Dulbecco’s Modified Eagle Medium (DMEM) medium supplemented with 10% fetal bovine serum (FBS). MCF-10A was maintained in DMEM/F12 medium supplemented with 10% FBS, insulin (0.01 mg/mL), epidermal growth factor (EGF; 0.02 mg/mL), and hydrocortisone (0.05 mg/mL). Both media contained 100 units/mL of penicillin and 100 μg/mL of streptomycin. The cell cultures were kept at 37 °C in a humidified atmosphere of 5% CO_2_ in air.

### Cell viability assay

The cell viability assay was performed using MTT^29^. Briefly, cell cultures were treated with different concentrations of curcumin (1.0, 5.0, 10.0, 25.0, 50.0, 75.0, and 100.0 µM), melphalan (0.5, 2.5, 5.0, 12.5, 25.0, 37.5, 50.0, 75.0, and 100.0 µM), and curcumin plus melphalan (5.85 + 46.98 µM, 11.65 + 93.95 µM, 17.47 + 140.93 µM, 23.29 + 187.90 µM, and 29.11 + 234.88 µM) or control medium for 24 and 48 h, respectively. After treatment, cells were washed with phosphate buffered saline (PBS) and incubated for 3 h in 0.5 mL of MTT solution (0.5 mg/mL in PBS) at 37 °C in 5% CO_2_ in an incubator. Next, the medium was removed and DMSO (0.5 mL) was added to each well to dissolve the resulting formazan crystals. The absorbance was measured at a wavelength of 595 nm. The cell viability of MCF-7, MDA-MB-231, and MCF-10A cells cultured in the presence of the assessed compounds was calculated as a percentage of the control cells, and the IC_50_ values were obtained from dose–response curves. All experiments were performed in triplicate, and the IC_50_ was calculated using GraphPad Software 6.0 (GraphPad Inc., San Diego, CA, USA). A Trypan blue exclusion assay was also performed to assess the effect of curcumin, melphalan, and CURC/MEL on cell viability. MDA-MB-231 cells were treated as described before, washed with PBS, and resuspended with 100 µL of trypsin in 500 µL of DMEM with 2% FBS. An aliquot was stained with Trypan Blue dye (1:1) and the viable cells were immediately counted in a Neubauer chamber using an optical microscope. The results are expressed as a percentage of viable cells relative to untreated controls.

### Selectivity index (SI) calculation

The selectivity index (SI) was calculated as the ratio of non-tumorigenic cell MCF-10A IC_50_ to the IC_50_ of the cancer cell lines MCF-7 or MDA-MB-231, as described by Passos et al.^30^ with modifications.

### Clonogenic assay

The clonogenic assay was performed using crystal violet^31^. MDA-MB-231 cells were adjusted to a density of 10^3^ cells/per well and incubated in a 12-well plate for 24 h. The cells were treated with curcumin (23.29 µM or 11.65 µM), melphalan (187.90 µM or 93.95 µM), CURC/MEL (11.65 + 93.95 µM), or control medium for 24 h and the medium was replaced every three days. After 18 days, colonies were fixed in methanol (Sigma, St. Louis, MO, USA) for 10 min and stained with 5% crystal violet (Vetec; Rio de Janeiro, RJ, Brazil) for 30 min at room temperature. For colony analysis, colonies were washed five times with PBS for 5 min and 50 cells were counted using an Eclipse TS100 inverted microscope (Nikon, Tokyo, Japan).

### Detection of reactive oxygen species (ROS)

Detection of reactive oxygen species (ROS) was performed using MDA-MB-231 cells adhered to 96-well opaque culture plates and treated with curcumin (23.29 µM or 11.65 µM), melphalan (187.90 µM or 93.95 µM), CURC/MEL (11.65 + 93.95 µM), or control medium for 3 h. The cells were then stained with 10 μM 2′-7′-dichlorodihydrofluorescein diacetate (DCFH-DA, Sigma-Aldrich) for 30 min in the dark, and ROS was measured immediately at 500/526 nm excitation/emission wavelengths using a BD FACSCalibur™ flow cytometer (Becton Dickinson, Franklin Lakes, NJ, USA) and analyzed using BD CellQuest™ Pro software (Becton Dickinson).

### Cell cycle analysis

MDA-MB-231 cells were treated with curcumin (23.29 µM or 11.65 µM), melphalan (187.9 µM or 93.95 µM), CURC/MEL (11.65 + 93.95 µM) or control media for 24 and 48 h, respectively. After treatment, the cells (5 × 10^5^) were washed with PBS and fixed in 70% (v/v) ice-cold methanol/PBS for at least 1 h at 4 °C. The fixed cells were washed once with PBS and then incubated in PBS supplemented with 10 μg/mL propidium iodide (PI) and 20 μg/mL RNAse at 37 °C for 30 min at room temperature in the dark. For each sample, 10,000 events were analyzed using a BD FACScan™ analyzer (Becton Dickinson) and BD CellQuest™ Pro software^21^.

### Annexin V binding assay

Double staining for annexin V-fluorescein isothiocyanate (FITC) and PI was performed with the Annexin-V apoptosis detection kit (Molecular Probes, Eugene, OR, USA). MDA-MB-231 cells were treated with curcumin (23.29 µM or 11.65 µM), melphalan (187.9 µM or 93.95 µM), CURC/MEL (11.65 + 93.95 µM) or control medium for 24 and 48 h, respectively. Cells were then washed twice in cold annexin V-buffer and centrifuged at 2000 rpm for 5 min. Pellets were resuspended in 20 μL of annexin V FITC, and after 15 min of incubation in the dark, 480 μL of annexin V-buffer containing 0.5 mg/mL PI was added according to the manufacturer’s instructions. Annexin V-FITC labeling was recorded on a BD FACSCalibur™ platform (Becton Dickinson) and analyzed using BD CellQuest™ Pro software.

### Western blotting

For preparation of protein extracts, after treatment for 24 h, cells were washed with PBS and lysed in liquid nitrogen. Cells were then scraped using lysis buffer (5 mM Tris–HCl, 10 mM ethylenediaminetetraacetic acid, 5 mM sodium fluoride, 1 mM sodium orthovanadate, 1 mM phenylarsine oxide, 1 µM okadaic acid, and 1 mM phenylmethylsulfonyl fluoride; pH 7.4) and protease inhibitor cocktail (Sigma-Aldrich). The lysate was collected, sonicated, and cleared by centrifugation for 5 min at 4 °C and the supernatant was stored at − 80 °C. Equal amounts of total cellular proteins (80 µg) were loaded on sodium dodecyl sulfate–polyacrylamide gel electrophoresis (SDS-PAGE) and transferred into polyvinylidene difluoride (PVDF) membranes (Immobilon P, Millipore, Burlington, MA, USA). Membranes were blocked for 2 h in Tris-buffered saline containing 1% Tween 20 (TBS-T) and 5% nonfat milk and incubated for 2 h with the primary antibody (1:1000), then washed with TBS-T and incubated with a peroxidase-conjugated secondary antibody (1:1000) for 2 h. Protein bands were visualized with the enhanced chemiluminescence (ECL) kit (Amersham, UK) using C-DiGit Chemiluminescent Western Blot Scanner (LI-COR Biotechnology, Lincoln, NE, USA). Images were analyzed using ImageJ 1.51p software (National Institutes of Health, Bethesda, MD, USA) and results were expressed as arbitrary units, calculated as the fraction of pixels measured in each band relative to the β-actin bands^32^. Anti-p53 (DO-1) antibody was purchased from Santa Cruz Biotechnology (Santa Cruz, CA, USA). Anti-p21 (2947) and Anti-β-actin (1978) antibodies were purchased from Sigma-Aldrich. Anti-caspase-3 (#9662), anti-caspase-7 (#9492), anti-caspase-9 (#9502), and PARP (#9542) antibodies were purchased from Cell Signaling Technology (Danvers, MA, USA) Supplementary Information.

### Immunocytochemistry

The cells were treated with curcumin (23.29 µM or 11.65 µM), melphalan (187.90 µM or 93.95 µM), CURC/MEL (11.65 + 93.95 µM) or control medium for 24 h. Next, the cells were fixed with 4% paraformaldehyde, 0.3% bovine serum albumin (BSA), and 0.5% Triton X-100 for 20 min and permeabilized with Triton X-100 (0.5% in PBS) for 25 min. The cells were then washed in PBS three times and incubated with primary antibodies for apoptosis markers (anti-caspase-3, anti-caspase-7, and anti-caspase-9; Cell Signaling Technology) in the dark for approximately 1 h. Next, cells were washed in PBS three times and incubated with the fluorescence secondary antibody (Alexa 647, ThermoFisher, Waltham, MA, USA) in the dark for approximately 1 h. The cells were washed and the fluorescence collected by flow cytometry on a BD FACSCalibur™ platform (Becton Dickinson) was analyzed using BD CellQuest™ Pro software.

### Statistical analysis

Data were analyzed using Student’s *t* test when comparing two groups or one-way analysis of variance (ANOVA) for more than two groups using GraphPad Prism 6.0 software. Data were considered to be statistically significant at P < 0.05.

### Supplementary Information

Supplementary Information.

## Data Availability

The datasets generated during and analyzed during the current study are available from the corresponding author on reasonable request.
