# Supplementary material for: Curcumin and melphalan cotreatment induces cell cycle arrest and apoptosis in MDA-MB-231 breast cancer cells
Source: Sci Rep. 2023 Aug 18;13:13446. doi: 10.1038/s41598-023-40535-5 (PMC10439215; doi:10.1038/s41598-023-40535-5)
Supplement: Supplementary file 1 — Supplementary Information. [file 41598_2023_40535_MOESM1_ESM.doc]

**Curcumin and melphalan cotreatment induces cell cycle arrest and apoptosis in MDA-MB-231 breast cancer cells**

Carlos Luan A. Passos^a☯^, Renata Madureira Polinati^a☯^, Christian Ferreira^a☯^, Nathalia Alexia N. dos Santos^a^, Daniel Galinis V. Lima^a^, Jerson Lima Silva^b^, Eliane Fialho^a*^

^a^ Functional Foods Laboratory, Nutrition Institute, Federal University of Rio de Janeiro, Rio de Janeiro, Brazil.

^b^ Medical Biochemistry Institute, Federal University of Rio de Janeiro, Rio de Janeiro, Brazil.

^☯^These authors contributed equally to this work.

^*^Correspondence to: Profª. Eliane Fialho de Oliveira, PhD. Departamento de Nutrição Básica e Experimental, Instituto de Nutrição Josué de Castro, Centro de Ciências da Saúde, Universidade Federal do Rio de Janeiro, UFRJ, Caixa Postal 68041, Cidade Universitária, Ilha do Fundão, Rio de Janeiro, CEP 21941-590, Brazil. E-mail adress: fialho@nutricao.ufrj.br. Fax number: + 55 21 2280 8343. Tel number: +55 21 3938 6799.


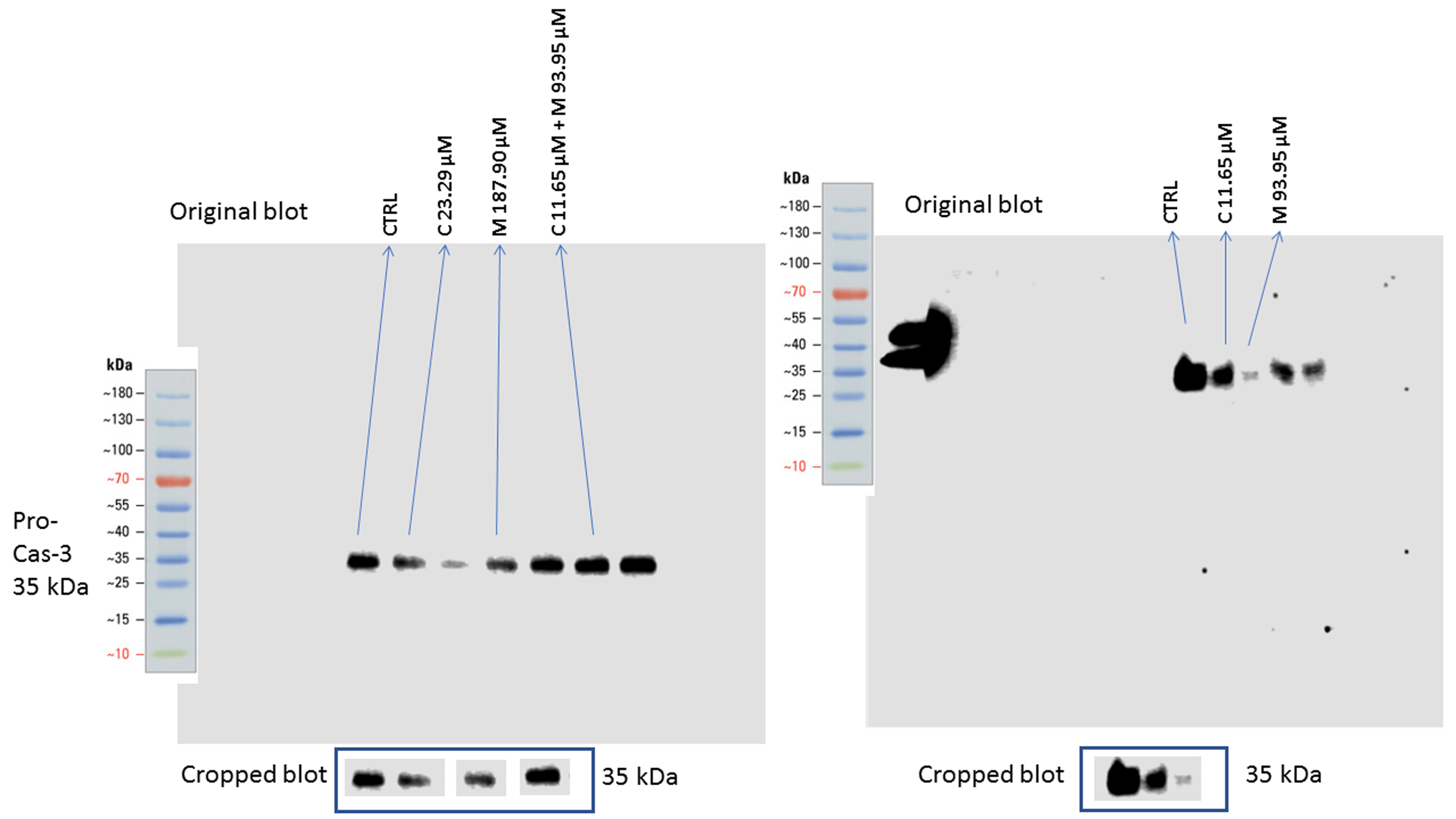


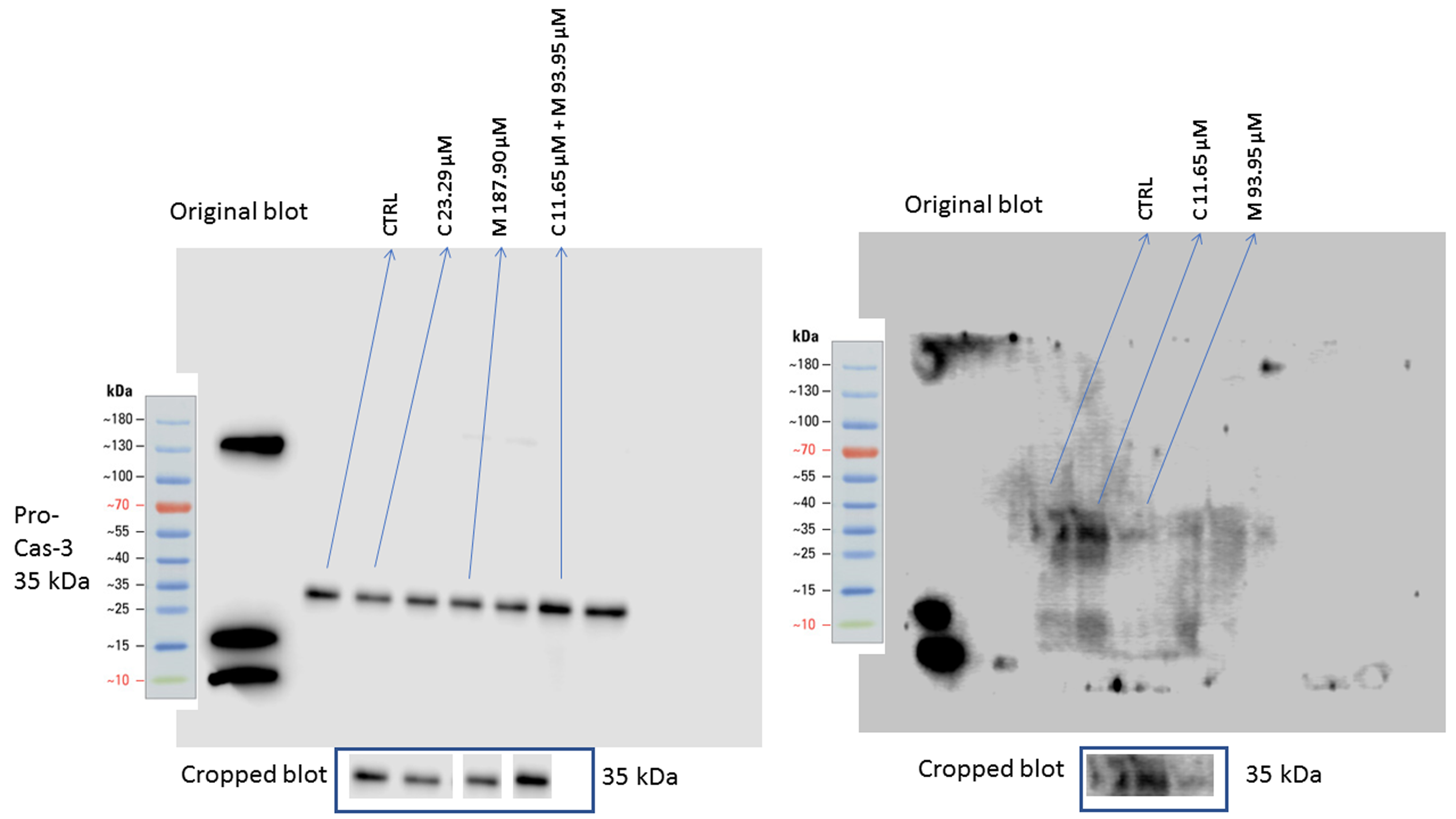


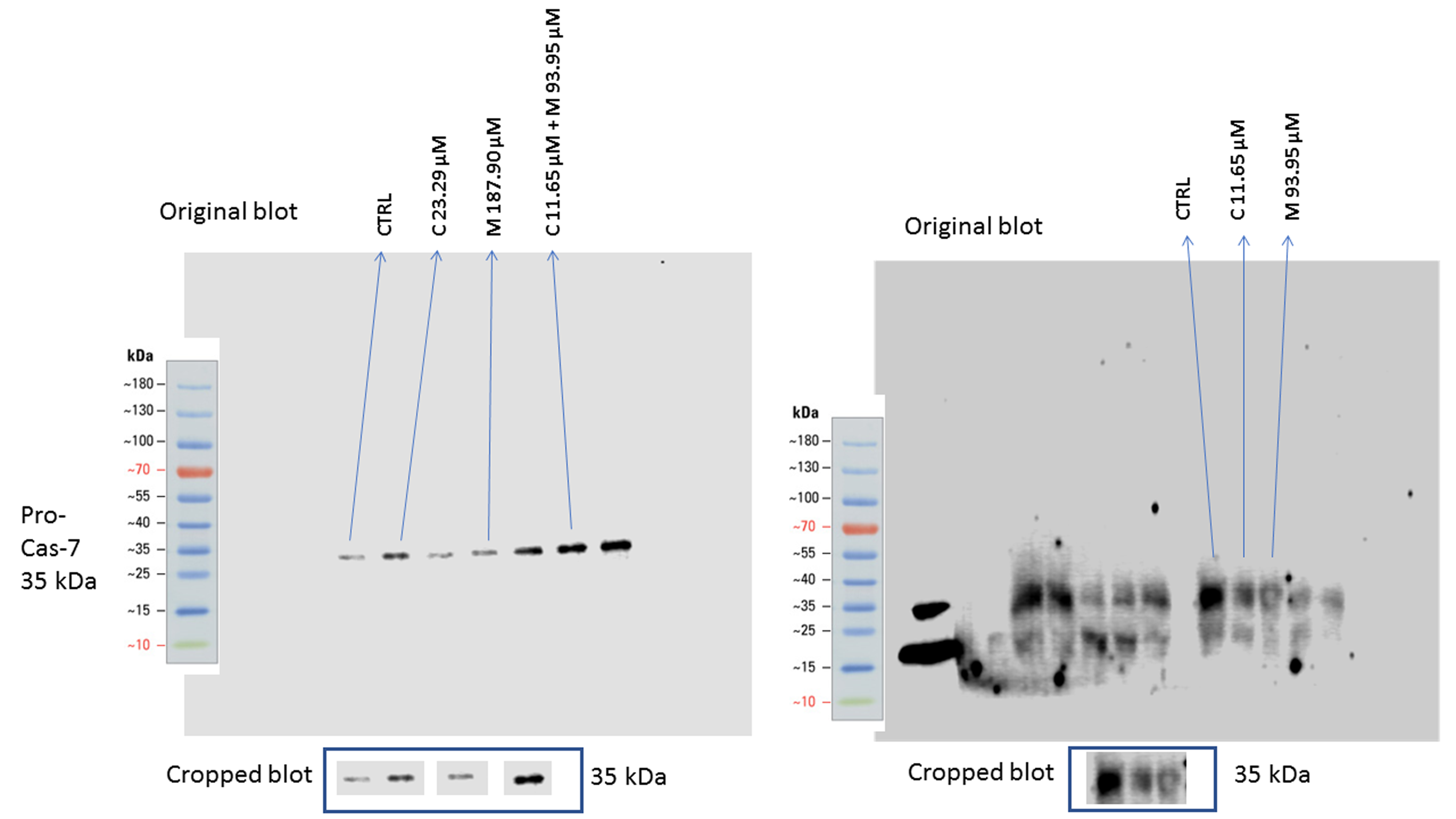


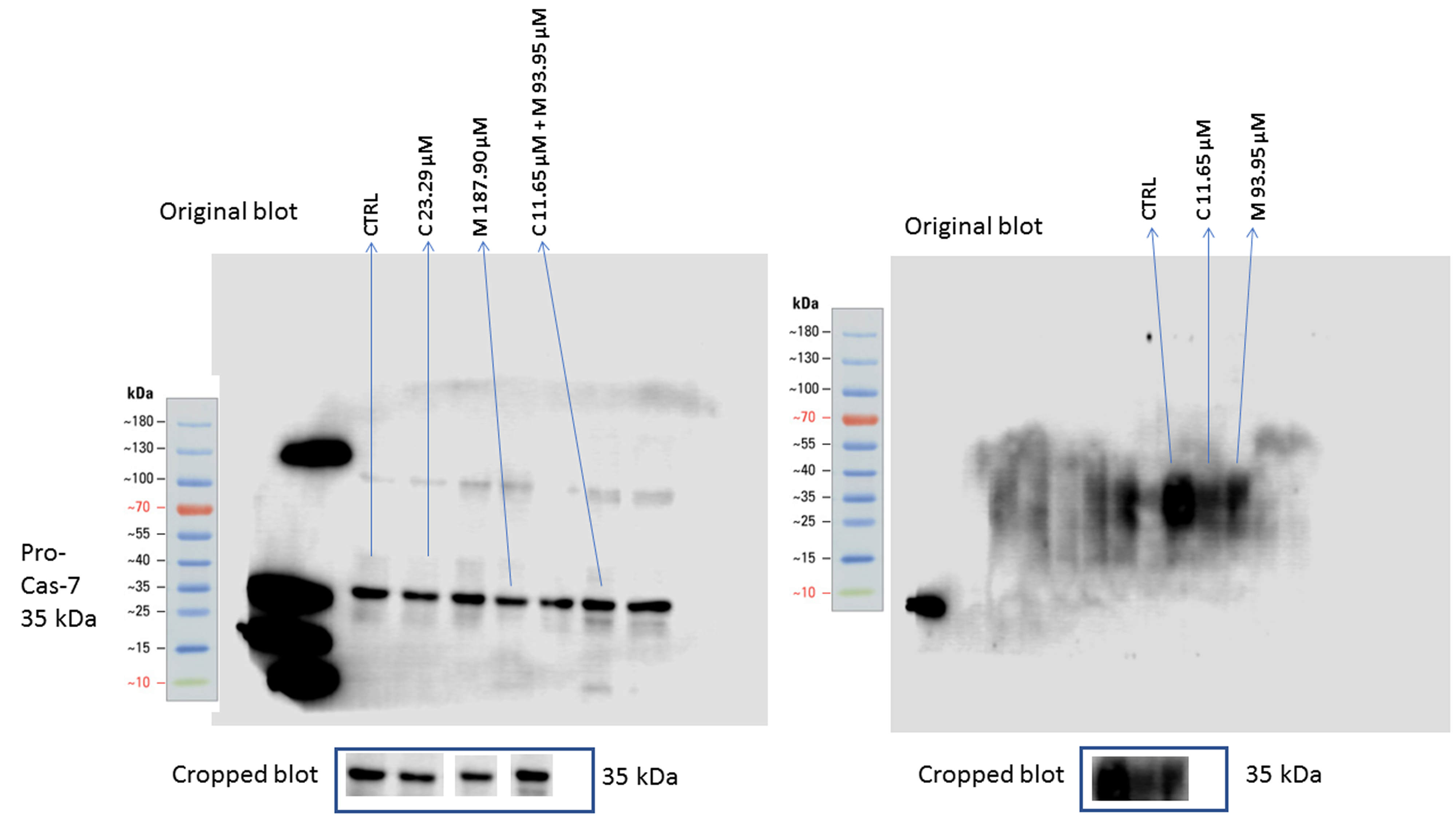


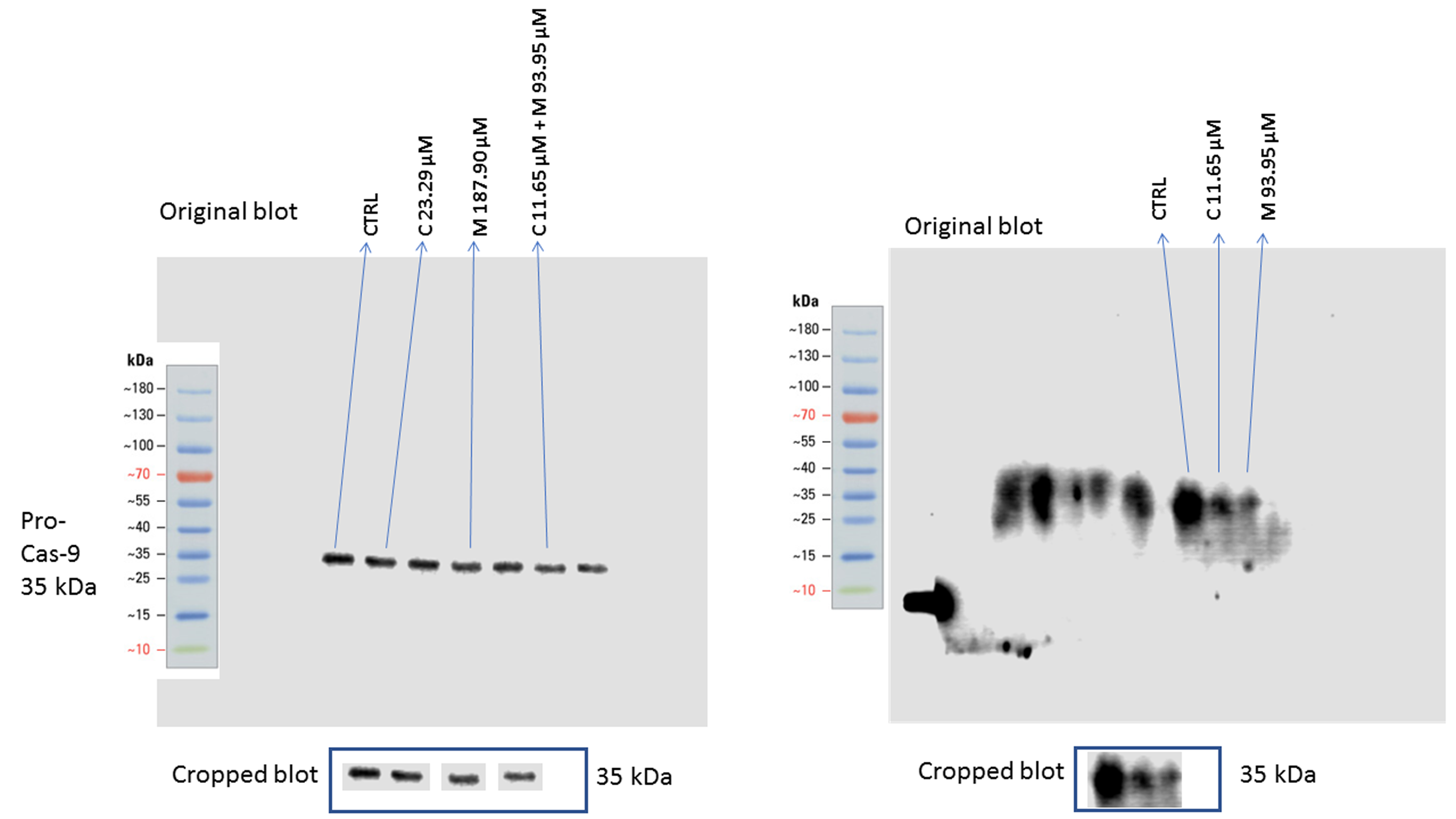


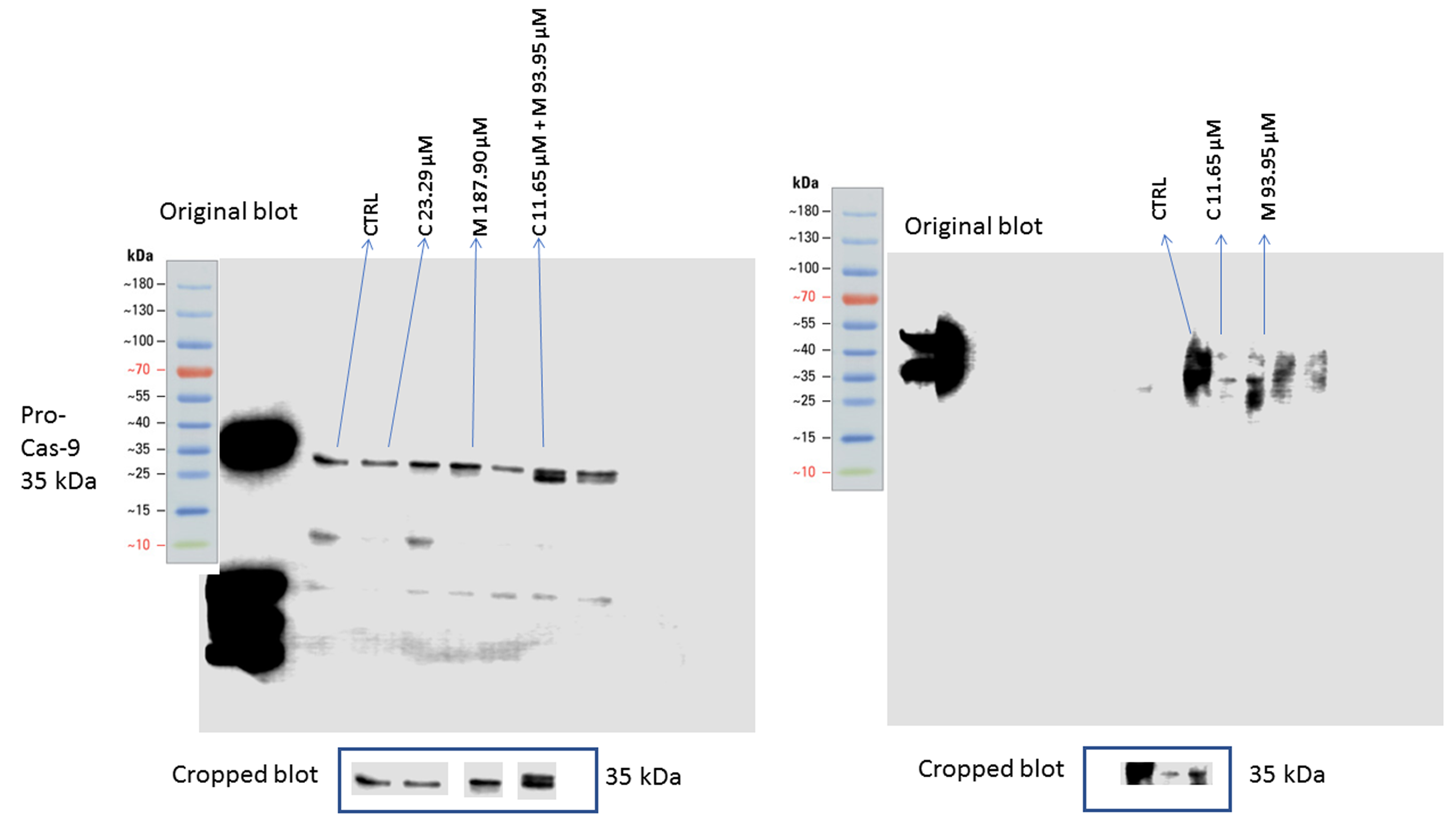


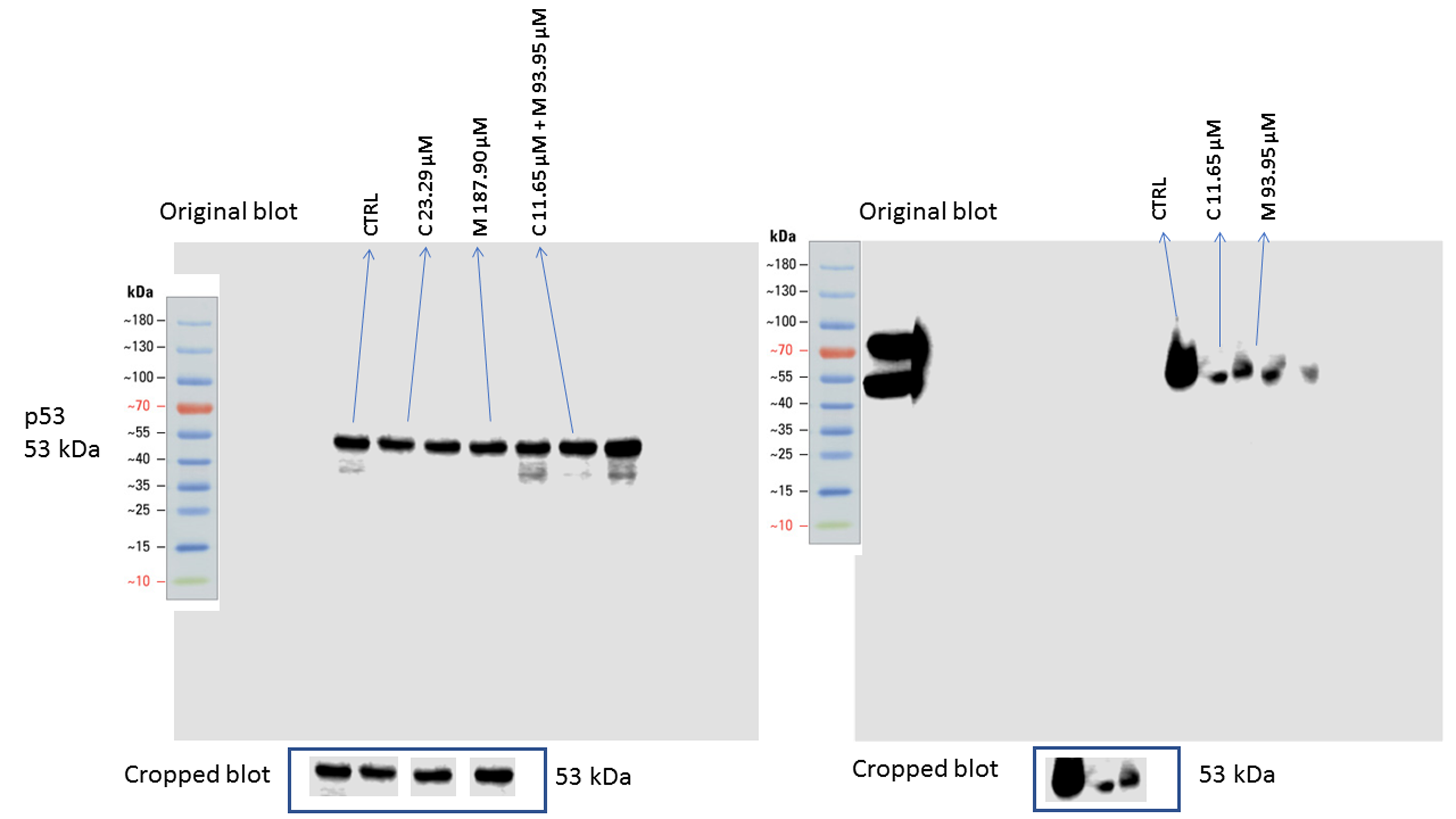


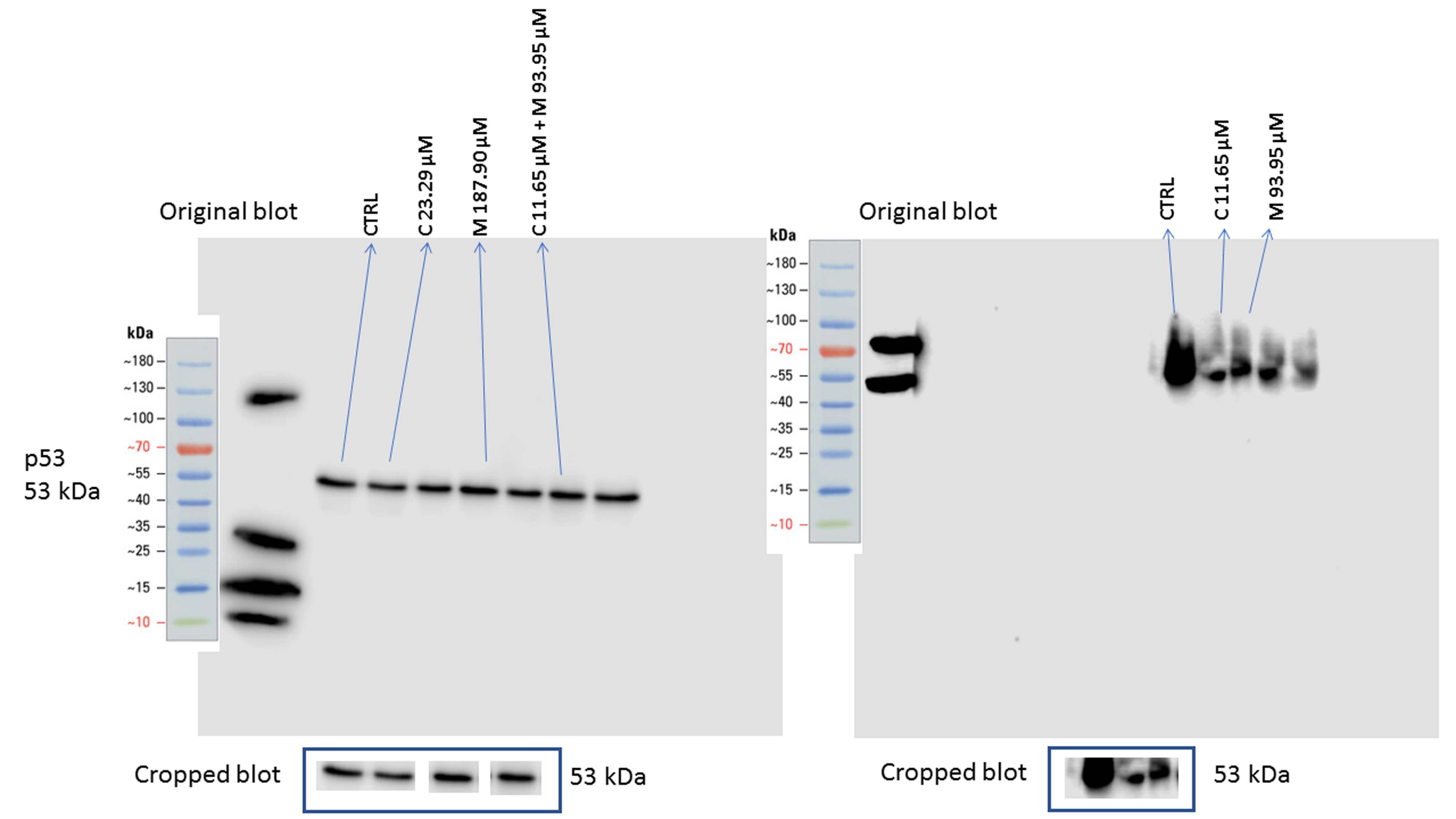


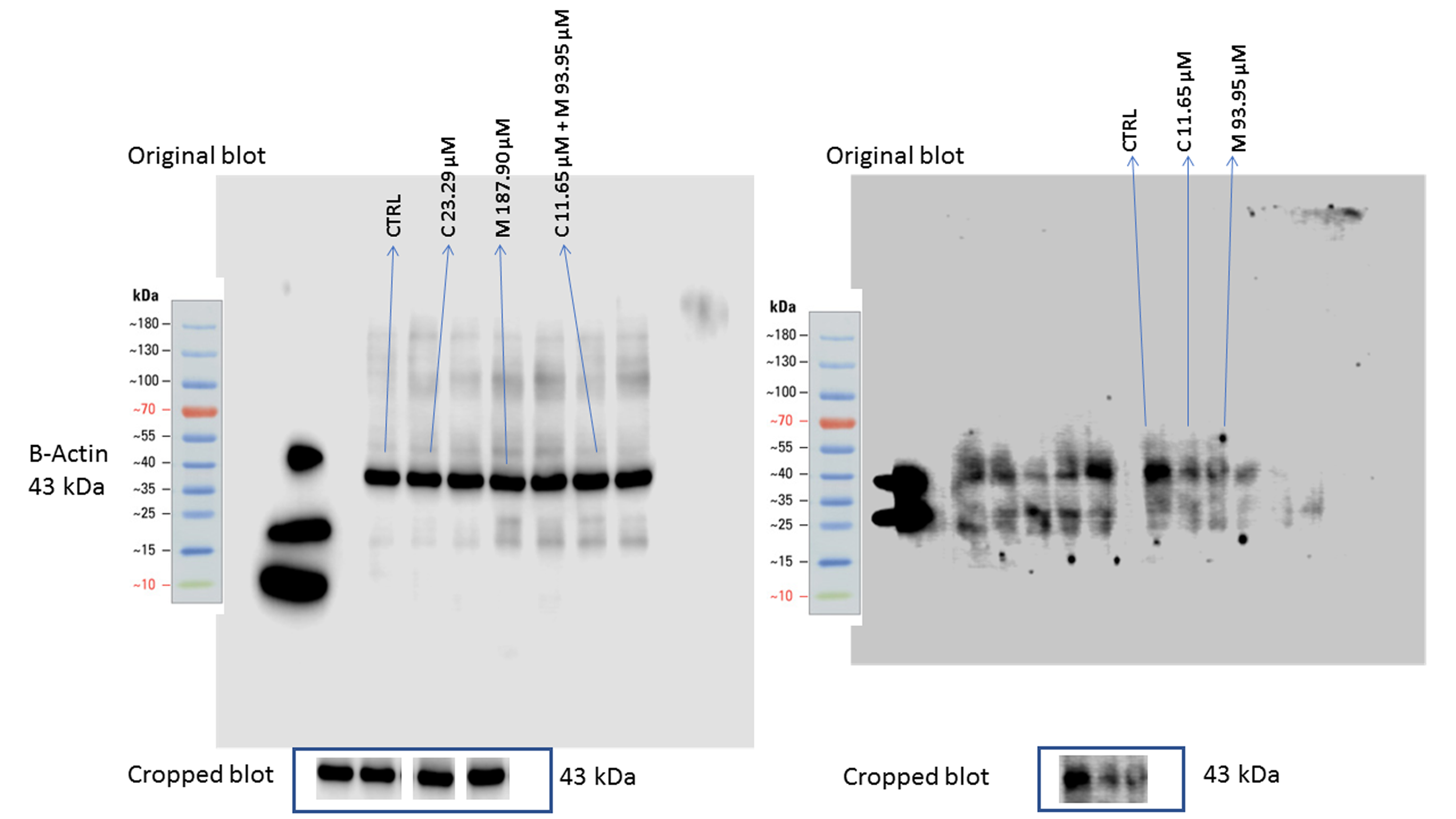


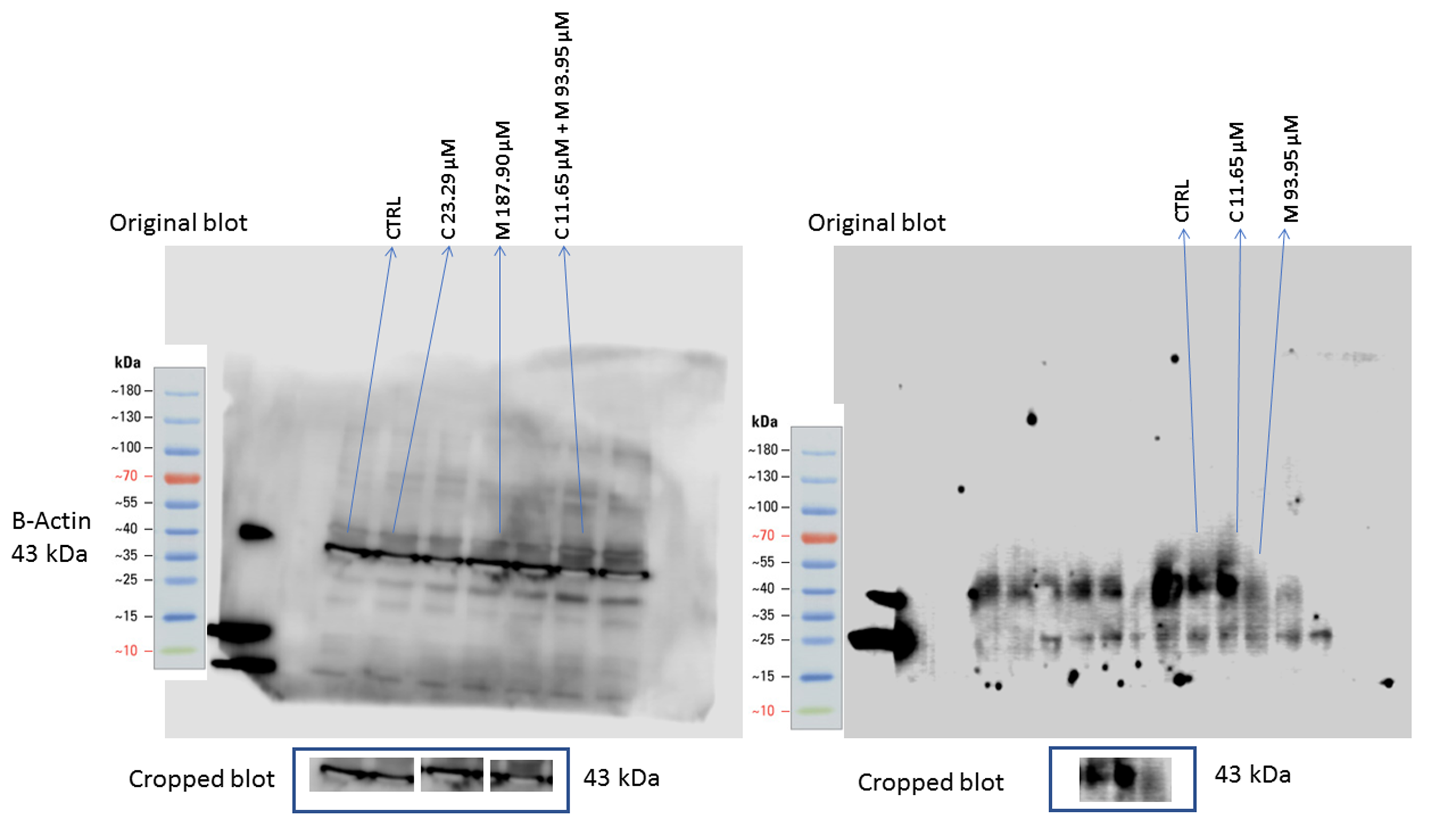


**Supplementary Figure 1.** The original Western Blots from **Figure 5.**
